# Impact of Inconsistent Policies for Transfusion-Transmitted Malaria on Clinical Practice in Ghana

**DOI:** 10.1371/journal.pone.0034201

**Published:** 2012-03-27

**Authors:** Alex K. Owusu-Ofori, Imelda Bates

**Affiliations:** 1 Department of Clinical Microbiology, Komfo Anokye Teaching Hospital, Kumasi, Ghana; 2 Disease Control Strategy Group, Liverpool School of Tropical Medicine, Liverpool, United Kingdom; Queensland Institute of Medical Research, Australia

## Abstract

**Background:**

Policies concerning the prevention of transfusion transmitted malaria (TTM) are the responsibility of blood transfusion services and malaria control programmes. To prevent spreading drug resistance due to over-use of malaria drugs, recent malaria treatment guidelines recommend prompt parasitological confirmation before treatment is started. In contrast, blood safety policies from the World Health Organisation (WHO) recommend presumptive malaria treatment for recipients of blood in endemic countries but evidence supporting this approach is lacking. Our study documented how these conflicting policies relating to malaria transmission through blood transfusion impact on clinical practice in a teaching hospital in West Africa.

**Methods/Principal Findings:**

We randomly selected and reviewed case notes of 151 patients within 24 hours of their receiving a blood transfusion. Transfusion practices including the confirmation of diagnosis and anti-malarial treatment given were compared across three departments; Obstetrics and Gynaecology (O&G), Paediatrics and Medicine. Overall, 66 (44%) of patients received malaria treatment within 24 hrs of their blood transfusion; of which only 2 (3%) received anti-malarials based on a laboratory confirmation of malaria. Paediatric patients (87%) received the most anti-malarials and only 7% and 24% of recipients in medicine and O&G respectively received anti malarials. In 51 patients (78%), the anti-malarials were prescribed at the same time as the blood transfusion and anti-malarials prescriptions exceeded the number of patients with a presumptive diagnosis of malaria.

**Conclusions:**

It is common practice in paediatrics to prescribe anti-malarials routinely with blood transfusions. This contravenes the malaria treatment guidelines of laboratory confirmation before treatment but is in accordance with the less-well evidenced blood safety guidelines. There is an urgent need for more evidence about the clinical impact of transfusion transmitted malaria to enable malaria and blood transfusion programmes to harmonize their policies and give clear guidance to clinicians who prescribe blood transfusions in malaria-endemic areas

## Introduction

Malaria is one of the most important parasitic diseases in the world. In 2009, there were approximately 225 million malaria cases in the world with 781,000 deaths [1]. Current measures to reduce the burden of malaria include insecticide- treated mosquito nets, intermittent preventive treatment, indoor residual spraying and malaria vaccines. An area of malaria control which has been neglected in endemic countries is malaria transmission through blood transfusion. Although blood transfusion has been recognised as one of the transmission routes for malaria since 1911 [2] the prevalence of transfusion transmitted malaria (TTM) in sub-Saharan Africa remains unknown. In endemic countries a substantial proportion of the population have asymptomatic parasitaemia. This makes it difficult to be sure whether malaria occurring after blood transfusion was acquired from the transfusion or not.

There are no evidence-based international guidelines for the prevention of TTM in sub-Saharan Africa and there is lack of harmonisation between policies produced by blood safety programmes and policies by malaria programmes. The World Health Organisation (WHO) recommends that donated blood should be tested for malaria “where appropriate and possible”, [3] but there is currently no method for screening blood for low-level parasitaemia that is sensitive, practical and affordable for use by transfusion services in endemic countries [4]. Microscopy is the most commonly used test but it is time-consuming and insufficiently sensitive [5]. Other transfusion guidelines suggest that transfusion recipients should be given prophylaxis with anti-malarials [6]. In the past, administration of anti-malarials such as chloroquine to recipients of blood transfusions was a widespread practice. Resistance to chloroquine means that it has largely been replaced by the more expensive artemisinin-based therapies and this has increased the cost of treating all transfusion recipients with anti-malarials by 5–7 fold making this practice unaffordable on a wide scale [7], [8]. In contrast to blood safety policies, malaria policies recommend that malaria treatment should only be prescribed on the basis of laboratory confirmation of infection [9] in order to reduce the development of drug resistance.

There are therapeutic dilemmas related to the use of anti-malarials and antibiotics in association with blood transfusions which are particularly apparent in paediatric practice. Anti-malarials are given presumptively on the basis that most febrile children have underlying malaria and that they are at high risk of transfusion-transmitted malaria. In the case of antibiotics the presumptions are that non-malaria infections are common in febrile children and that post-transfusion fever is frequently due to bacterial infections.

There is very little information in the literature about how African countries have incorporated WHO's recommendations concerning transfusion-transmitted malaria into their national policies and how these policies have been translated into practice. The Democratic Republic of Congo, Comoros and Malawi are carrying out some screening of donated blood for malaria using microscopy [10]. Other countries such as Nigeria and Rwanda do not have any policy for reducing TTM and a survey of seven sub-Saharan Francophone African countries found that none of them were systematically screening blood donors for malaria [11]. In the absence of clear national policies clinicians may rely on international recommendations which may not be applicable in their setting or they use individualised practices that are not based on evidence.

Ghana's malaria policy recommends laboratory confirmation of malaria before starting treatment in all situations except for children under 5 years and for suspected severe malaria if laboratory confirmation is not immediately possible. The first line treatment for uncomplicated malaria which is the only type likely to be present in clinically well blood donors is oral artesunate-amodiaquine [12]. As in many other countries in sub-Saharan Africa, the blood transfusion policy in Ghana makes no mention of screening donated blood for malaria or of treating recipients prophylactically. The aim of our study was to document how these conflicting or absent policies relating to malaria transmission and blood transfusion impact on clinical practice in a teaching hospital in West Africa. Analysis of discrepancies between policies and practice would enable us to identify priorities for better harmonisation of policies and to identify gaps in the evidence regarding TTM.

## Methods

Approval was given for this study by 2 ethics committees: the Liverpool School of Tropical Medicine, Research Ethics Committee in Liverpool, UK and the Committee on Human Research Publication and Ethics (CHRPE) of the School of Medical Sciences, Kwame Nkrumah University of Science and Technology in Kumasi, Ghana.

This study was conducted at the Komfo Anokye Teaching Hospital (KATH) in the Ashanti region of Ghana between October and November 2009. KATH is the second largest hospital in Ghana and the referral centre for the middle and northern parts of the country. It has a 1000 bed capacity and carries out about 14,500 whole blood transfusions per year. There are no hospital based guidelines for the clinical use of blood or any written policies or protocols concerning transfusion transmitted malaria. The blood bank has transfusion monitoring forms which are given to ward staff when the blood is issued and are used for reporting any transfusion reactions.

Transfusion recipients from the departments of Obstetrics and Gynaecology, Paediatrics and Medicine were included in the study. These departments were selected because they use the majority (66%) of blood in the hospital. All patients in these departments who had received a transfusion within the previous 24 hours were eligible for the study. Exclusion criteria were untraceable case notes, transfer to a department not included in this study or more than one transfusion within the 24 hour time period. Each day the blood bank staff provided AOO with the names of two blood transfusion recipients in each of the three departments. The recipients were selected randomly from the list of patients who had received blood in the preceding 24 hours. On six occasions during the two month study a department had less than two transfusion recipients in 24 hours and so the name of a replacement participant from another department was provided by the blood bank staff. On one occasion the case notes of a patient from obstetrics could not be traced and as no other obstetric patient was eligible, a replacement was recruited from paediatrics.

At recruitment, a data collection form was used to extract information from the clinical notes of the transfusion recipients. The form was adapted from the KATH transfusion monitoring form, which is based on the national guidelines for the clinical use of blood in Ghana. The form was expanded to include information about the patient's diagnoses, laboratory tests (such as malaria microscopy, pre-transfusion haemoglobin) and treatment type (such as anti-malarials, antibiotics, diuretics). Information was also collected on monitoring of vital signs, duration of transfusion and documentation of adverse events of transfusion.

Predictive Analytics Software (PASW®) statistics 18 package (SPSS Inc, USA) was used to calculate frequency statistics including measures of central tendency and dispersion and the results were compared between the three departments. The practices relating to the prevention and treatment of malaria associated with transfusion were compared with known policies.

## Results

151 transfusion recipients were recruited into the study from medicine (41, 27.2%), obstetrics (51, 33.7%) and paediatrics (59, 39.1%). The majority of patients (64.9%) were female, because of the inclusion of the obstetric department, and the median age of all patients was 22.0 years (IQR: 4.0–36.0) (Table 1). 25 patients (16.6%) had received a blood transfusion during a previous admission and the highest rates of previous transfusions were in the department of medicine (45.5%). 66% of transfusion recipients from medicine, 65% from obstetrics and 15% from paediatrics had received a transfusion during the current admission prior to enrolment in the study. The mean pre-transfusion haemoglobin in all the transfusion recipients in the study was 4.5 g/dl (SD±1.2) (Table 1). All patients received either whole blood or packed red cells. The mean duration of transfusion for all study participants was 2.6 hours and was longest for paediatric transfusions (3.3 hours). Ninety four percent of all transfusions were completed (Table 1). There were 10 deaths (7%) among recipients within 24 hours of transfusion but none of these were attributed to the blood transfusions.

**Table 1:** Characteristics and practices among transfusion recipients within three departments in a Ghanaian Teaching Hospital.

	All (N = 151)	Medicine (N = 41)	Departments Obstetrics (N = 51)	Paediatrics (N = 59)
Median age (IQR)yrs	22.0 (4.0–36-0)	41.0 (29.5–56.0)	28.0 (22.0–38.0)	3.0 (1.0–6-0)
Completed transfusion (%)	142 (94.0)	36 (87.8)	51 (100)	55 (93.2)
>1 transfusion received (%)	82 (54.3)	27 (65.9)	33 (64.7)	9 (15.3)
Mean pre-transfusion haemoglobin ±SD (g/dl)	4.5±1.2	4.4±1.7	4.7±1.2	4.3±0.9
Mean duration of transfusion ±SD (hrs)	2.6±1.2	2.2±1.1	2.0±0.8	3.3±1.0
Documentation of transfusion reactions	13 (8.6)	4 (9.8)	5 (9.8)	4 (6.7)
Death within 24 hrs of transfusion (% )	10 (6.6)	6 (14.6)	2 (3.9)	2 (3.3)

*O*&G represents Obstetrics and Gynaecology; IQR represents interquartile range; SD represents standard deviation.

Sixty six (44%) patients received malaria treatment within 24 hours of their blood transfusion. There was marked variation in the use of anti-malarial drugs between departments with 51 (87%) children receiving malaria treatment compared to 12 (24%) obstetric patients and 3 (7%) adult medical patients (Table 2). In 51 of these patients (78%) the anti-malarials were prescribed at the same time as the blood transfusion. 84% (43/51) of those who received anti-malarials with the transfusion were children and only one had parasitaemia confirmed by microscopy. Eight patients had received anti-malarials at least 24 hours prior to transfusion and in 3 patients the timing of the anti-malarials was unknown. The temperatures charts for all transfusion recipients revealed that 34 (23%) developed fever in the 24 hours following transfusion. Post-transfusion fever was a particular problem in obstetric (24%) and paediatric (32%) transfusion recipients. Four transfusion recipients with post-transfusion fever received anti-malarials because it was thought that malaria was the cause of the fever. There was however only one malaria case confirmed on a blood film (Table 2). Information from patients' case notes indicated that clinically-suspected malaria was a reason for hospitalisation in 50 (30%) of the transfusion recipients; 70% of these were children. Within the department of paediatrics, a clinical diagnosis of malaria was made more frequently in younger children (Table 3). Overall 20 transfusion recipients (13%), 10/59 (17%) children, 3/51 (6%) obstetric patients and 7/41 (17%) medical patients, received malaria treatment with their transfusion or within 24 hours post-transfusion despite having no underlying clinical diagnosis of malaria, and 64 (42%) transfusion recipients were treated for malaria without any laboratory confirmation of parasitaemia. Quinine (40% of all anti-malarial prescriptions) and artesunate-amodiaquine (33%) were the most commonly prescribed anti-malarial drugs and these were used exclusively by the department of paediatrics. Quinine, which is recommended for severe malaria, was used more frequently in younger children (Table 3). The only anti-malarials prescribed in the departments of medicine and obstetrics and which formed 27% of all prescribed anti-malarials was artemeter-lumefantrine. No chloroquine or sulphadoxine-pyrimethamine was prescribed for any transfusion recipient.

**Table 2:** Practices related to transfusion transmitted malaria in three hospital departments.

	All (N = 151)	Medicine(N = 41)	O&G(N = 51)	Paediatrics(N = 59)
	n (%)	n (%)	n (%)	n (%)
Clinical diagnosis of malaria in admission (%)	50 (30)	0 (0)	9 (18)	41* (70)
Anti-malarial use (%)	66 (44)	3 (7)	12 (24)	51 (87)
Type of anti-malarial given				
Quinine	26 (40)	0 (0)	0 (0)	26 (51)
Artemether-Lumefantrine	18 (27)	3 (100)	12 (100)	3 (6)
Artesunate-Amodiaquine	22 (33)	0 (0)	0 (0)	22 (43)
Time of prescribing anti-malarial				
With transfusion	51 (77)	1 (33)	7 (59)	43 (84)*
At least 24 hours before transfusion	8 (12)	1 (33)	1 (8)	6 (12)
Within 24 hours post- transfusion	4 (6)	0 (0)	3 (25)*	1 (2)
Unknown	3 (5)	1 (33)	1 (8)	1 (2)
Other drugs				
Furosemide (%)	60 (40)	2 (5)	6 (12)	52 (88)
Antibiotics use (%)	118 (78)	25 (61)	49 (96)	44 (75)
Post transfusion fever (%)	34 (23)	3 (7)	12 (24)	19 (32)

* confirmed by positive malaria microscopy in one case.

**Table 3:** A comparison of anti-malarial use among children with and without clinical features of malaria.

	Malaria (N = 41)	Non-malaria (N = 18)
Median age (IQR) years	2.0 (1.0–4.0)	5.0 (1.8–9.3)
Children <5 yrs (%)	34 (82.9)	10 (55.5)
Anti-malarial use (%)	41 (61.0)	10 (55.5)
Type of anti-malarial given (%)		
Quinine*	25 (61.0)	1 (10)
Artemether-Lumefantrine	1 (2.4)	2 (20)
Artesunate-Amodiaquine	15 (36.0)	7 (70)

* recommended for severe malaria.

Overall 118 transfusion recipients (78%) received antibiotics, with the highest usage in obstetrics (96%) (Table 2). 81% of antibiotics were prescribed prior to the blood transfusion. 40% of all transfusion recipients, predominantly paediatric patients (88%), had furosemide prescribed at the same time as the blood transfusion (Table 2). The reason for prescribing a diuretic was only documented in the notes of two patients both of whom had heart failure. In 96% of patients there was no indication of why furosemide was given.

## Discussion

In Ghana, as in many other malaria endemic countries in sub-Saharan Africa, blood for transfusion is not screened for malaria and there are no clear policies about whether or not anti-malarials should be prescribed presumptively with blood transfusions. 44% of all transfusion recipients in our study received malaria treatment with their blood transfusion (77%) or in the 24 hours following the transfusion (6%) but only two (3%) had parasitaemia confirmed on microscopy. This practice of routine prescription of anti-malarials with blood transfusions without laboratory confirmation of infection occurred predominantly in paediatrics. Many of the children had a clinical diagnosis of malaria so it is possible that the anti-malarials were prescribed presumptively for their suspected infection. However, in children, 98% of malaria drugs for which the timing was known, were prescribed simultaneously with the blood transfusion and the number of children prescribed anti-malarials exceeded the number with suspected malaria. This indicates that in paediatric practice anti-malarial drugs are prescribed routinely with blood transfusions.

Prescribing malaria treatment routinely for transfusion recipients in endemic areas reflects WHO guidelines [6] and the advice in several published papers [13]–[16]. In contrast, other papers recommend restricting malaria treatment to selected ‘at risk’ transfusion recipients such as neonates [17]–[19]. However, evidence that transfusion-transmitted malaria is a significant clinical problem in endemic areas is almost non-existent. One study has documented malaria parasitaemia occurring in recipients of malaria-positive transfusions in Sudan [20], but we have recently observed that the majority of transfusion recipients in Ghana who developed parasitaemia post transfusion did not acquire it from the blood transfusion as parasite genotyping indicated that malaria transmission only occurred in 2% of those who received a malaria-positive blood transfusion (unpublished data).

There have been no studies from endemic areas comparing presumptive versus targeted malaria treatment for transfusion recipients and there is very scanty evidence underpinning recommendations for presumptive malaria treatment of transfusion recipients. In contrast, current malaria treatment guidelines recommending that treatment should only be given to those with proven infections [9] are well-supported by evidence and are designed to slow down the emergence of P. falciparum resistance to artemisinin [21], [22]. There has been a rise in the proportion of febrile patients with laboratory confirmation of malaria from 5% in 2000 to 35% in 2009 [1]. Although patients with negative malaria tests are still being treated for malaria [23] a Ugandan study has demonstrated that it is possible to improve the rates of diagnostic malaria testing and to restrict malaria treatment to those with positive results [24].

Our study found that the restrictive malaria treatment policy is not implemented in the context of blood transfusions in a malaria endemic area. International guidelines do allow for presumptive treatment of malaria if laboratory confirmation is not available [9] but any facility that is able to offer blood transfusion will almost certainly have a laboratory capable of providing a definitive diagnosis of malaria. The practice of treating every fever presumptively for malaria is entrenched [25] and our study demonstrated that in practice, and particularly in paediatric practice, clinicians choose presumptive treatment for transfusion recipients rather than laboratory confirmation of malaria diagnosis prior to initiation of treatment. Implementation of the WHO recommendations to screen donated blood for malaria is hampered by a lack of suitable screening methods [4] which may partly explain the emphasis on presumptive treatment as an alternative strategy. Our study shows widespread prescribing of anti-malarials with transfusion especially in paediatric practice. These prescriptions are based on assumptions about the need for anti-malarials as prophylaxis against transfusion transmitted malaria or as treatment for malaria as a cause of anaemia. Quinine is the recommended treatment for severe malaria and was prescribed for the majority of children with clinically-diagnosed malaria (61%). Severe malaria requiring urgent treatment is a justification for initiating treatment for malaria without waiting for laboratory confirmation.

The mean duration of transfusion in children was longer than in adults and diuretics were prescribed with the blood transfusion for 88% of children. These practices do not appear to be consistent with any policies or guidelines but are based on assumptions that the symptoms of severe malarial anaemia, particularly respiratory distress, result from biventricular failure [26] and that these children are not hypovolaemic [27]. Recent evidence has shown that children with severe febrile illness and impaired perfusion do not benefit from fluid resuscitation [28]. Clinical guidelines [6] indicate that diuretics should only be used in transfusion recipients likely to develop or who develop cardiac failure. 88% of children in our study were given furosemide with their blood transfusion and in almost all cases there was no indication of why it was given.

The use of concomitant antibiotic in transfusion recipients ranged from 61–96% across the three departments and is likely to represent treatment for underlying conditions. In clinical practice in Africa it can be difficult to differentiate between severe malaria and sepsis, and both conditions may co-exist [29]. Among anaemic children in Malawi who were transfused for severe anaemia, 64% of them had malaria parasitaemia. In this study a pathogen was isolated from blood cultures in 24.7% of patients with post transfusion fever [30]. Recent data from Kenya [31] has shown an 8.8% bacterial contamination rate of paediatric whole blood, and may suggest that sepsis plays an important role in post transfusion fever. Ideally blood cultures should be taken before empirical antibiotics are started [32] but it is common for antibiotics and anti-malarials to be prescribed simultaneously [9], [33] and there is evidence that this approach may reduce mortality in severe malaria [34]. More studies are needed to establish the effects of bacterial contamination on transfusion recipients and the role, if any, of concomitant antibiotic use with transfusion.

**Figure 1:**
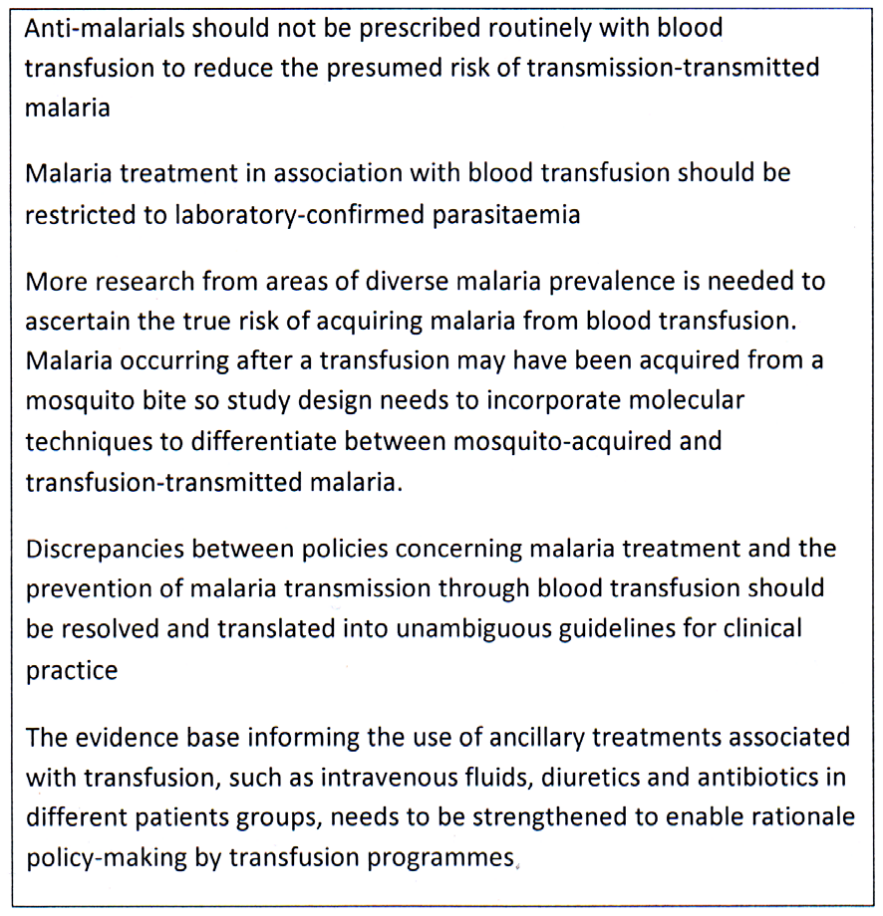
Recommendations concerning transfusion-transmitted malaria in malaria endemic countries in Africa. A summary of recommendations from the current situation of transfusion-transmitted malaria in malaria endemic countries in Africa.

These results of this study have enabled us to make several recommendations concerning transfusion-transmitted malaria (Figure 1). We have shown that where international malaria and transfusion policies are in conflict, such as restricting anti-malarials to proven infections versus presumptive treatment of transfusion recipients, clinicians choose to adhere to transfusion policies rather than malaria policies (Table 4) even though malaria policies have a much stronger evidence base than transfusion policies. Nevertheless, there is still widespread inappropriate use of anti-malarials in the context of blood transfusion in Africa. There is an urgent need for harmonised policy-making by malaria and blood transfusion programmes so that clinicians, and particularly paediatricians, receive clear messages about how to reduce malaria transmission through blood transfusions. This harmonisation needs to be preceded by research to generate better knowledge about the prevalence and clinical impact of transfusion transmitted malaria, and about the cost-effectiveness of various blood donor screening strategies taking account of the need to avoid exacerbating blood shortages by unnecessarily excluding donors. Our study has demonstrated that until these critical gaps in the evidence about transfusion-transmitted malaria are addressed and the relevant policies are harmonised, these mixed messages will continue to be translated into inconsistent management of patients receiving blood transfusions in malaria-endemic areas.

**Table 4:** Summary of policies and practices concerning transfusion transmitted malaria.

WHO recommendations	Policies in Ghana	Practice in KATH	Research knowledge needed about..
Donated blood should be tested for malaria [5]	All units to be tested for HIV I & II, Hepatitis B and C and syphilis and any other transfusion transmissible disease [35]	No malaria screening of donated blood	Screening methods for malaria that are practical and sensitive enough for use by transfusion services
In endemic areas, there is a high risk of transmitting malaria by transfusion. All transfusion recipients should receive routine treatment for malaria [6]	No policy on routine treatment for malaria	37% of transfusion recipients received anti-malarials with their transfusion or within 24 hours post-transfusion	Cost-effectiveness of malaria screening in different transmission zones
Anti-malarials should only be prescribed for proven malaria infections [9]	Confirm cases of malaria before initiating treatment (treatment can be started in severe cases but a confirmation from the laboratory is needed) [12]	3% (2/66) had a laboratory confirmation of malaria before treatment	Rates of malaria transmission by transfusion with genotyping to confirm that malaria was acquired through transfusion
			Effectiveness of presumptive malaria treatment of transfusion recipients compared to treatment restricted to proven infections
In patients at risk of circulatory overload, red cells are preferable to whole blood. Treat volume overload and cardiac failure with diuretics [6]	Diuretics should only be given in recipients with heart failure. For patients with heart failure lower the rate of transfusion [36]	88% of children were given diuretics	Effective approaches for ensuring that transfusion guidelines are evidence-based, regularly reviewed and implemented
